# Diminishing Effects After Recurrent Use of Self-Guided Internet-Based Interventions in Depression: Randomized Controlled Trial

**DOI:** 10.2196/14240

**Published:** 2019-10-02

**Authors:** Lara Bücker, Patricia Schnakenberg, Eirini Karyotaki, Steffen Moritz, Stefan Westermann

**Affiliations:** 1 Department of Psychiatry and Psychotherapy University Medical Center Hamburg-Eppendorf Hamburg Germany; 2 Department of Clinical Psychology VU Amsterdam Amsterdam Netherlands; 3 Idiographic Dynamics Lab Department of Psychology University of California Berkeley, CA United States

**Keywords:** eHealth, self-management, depressive symptoms, randomized controlled trial

## Abstract

**Background:**

Self-guided internet-based interventions have several advantages over guided interventions and are generally effective in treating psychiatric symptoms.

**Objective:**

We aimed to investigate whether the use of a new self-guided internet-based intervention (MOOD) would lead to a significant reduction in depressive symptoms compared with a care-as-usual (CAU) control group in a sample of individuals with depressive symptoms, most of whom had already used a different self-guided internet-based intervention in a previous trial.

**Methods:**

A total of 125 individuals were randomized to the intervention condition (MOOD) and received access to the intervention for a period of six weeks or a CAU group. After six weeks, all participants were invited to take part in the post assessment. The Beck Depression Inventory-II served as the primary outcome.

**Results:**

Both intention-to-treat as well as per-protocol analyses indicated that the depressive symptomatology decreased in both conditions but showed no advantage for those who had used MOOD. Subsequent moderation analyses suggested that those individuals who had less experience with psychotherapy benefitted to a greater extent compared with those with more experience.

**Conclusions:**

Self-guided internet-based interventions are deemed a suitable first-step approach to the treatment of depression. However, our results indicate that they are more efficacious in those with less psychotherapy experience.

**Trial Registration:**

ClinicalTrials.gov NCT03795480; http://clinicaltrials.gov/ct2/show/NCT03795480

## Introduction

### Background

Major depression (MD) is one of the most common mental disorders, with more than 300 million people affected worldwide [1]. MD has an aggregated lifetime prevalence of 10.8% and a 1-year prevalence of 7.2% [2], and it represents an enormous personal and economic burden [3,4].

There is evidence for the effectiveness of psychotherapy and pharmacotherapy in the treatment of depression [5-7]. Although classic face-to-face psychotherapy is effective, it is not possible to treat all affected individuals by this method. According to the World Health Organization (WHO), the treatment gap for depression is 56.3% [8]. This means that a large number of individuals suffering from depressive symptoms remain untreated. This treatment gap can be attributed to several different causes. One of the reasons being that there is an insufficient number of therapists, resulting in long waiting lists. In rural areas, psychologists and psychiatrists are especially underrepresented [9]. Furthermore, general practitioners often do not recognize MD, and misdiagnoses are frequent [10]. Besides these barriers on the supply side, there are also barriers on the demand side. These include patient fear about stigmatization, (expected) discomfort discussing one’s own mental health problems, the wish to overcome problems by oneself, a lack of awareness of the need for help, and the anticipated high cost of treatment [11,12]. In addition, it has been shown that greater levels of depression are associated with increased perceived barriers to seeking help, as depression is linked to deficient motivation and reduced activity, as well as to negative cognitive biases (eg, a negative view of the world or future) [13].

### The Potential of Internet-Based Interventions

Internet-based interventions can help overcome the supply and demand barriers of conventional face-to-face treatment. Internet-based treatments could reach people who fear stigmatization, are widely accessible (especially for those living in rural areas or those with physical barriers), and provide a high level of privacy [14,15]. Furthermore, the individuals themselves can decide when and where they want to use the intervention. In addition, these interventions are usually available at low cost and, thereby, are affordable for individuals with limited financial resources [16]. In recent years, numerous internet-based interventions have been developed and investigated for their feasibility, acceptance, effectiveness, and side effects, most being used in the treatment of depression and anxiety disorders [17]. To date, a wide range of studies has provided evidence of the effectiveness of internet-based interventions. Studies have found effect sizes ranging from small to large [18-25].

Internet-based interventions can be categorized as either unguided or guided. Guided internet-based interventions are supported by a therapist or a trained person (eg, via frequent email correspondence or telephone support). In self-guided internet-based interventions, the patient does not receive any additional human support. Meta-analyses have found similar effect sizes for guided internet-based treatments when compared with classical face-to-face treatment [18,26]. According to van Ballegooijen et al [27], the average percentage of completed sessions is similar for guided internet-based interventions when compared with face-to-face therapy, although more individuals complete all sessions of the face-to-face therapy. However, individuals who dropped out from face-to-face therapy only completed 24.5% of the intervention, whereas noncompleters of guided internet-based interventions completed on average 42.1%. Many other studies have shown that internet-based interventions often have high dropout rates [28]. A meta-analysis that examined predictors of treatment adherence in self-guided internet-based interventions for depression identified several demographic and psychopathological factors (male gender, low educational background, and comorbid anxiety symptoms) that predict dropout [29].

Another meta-analysis by Richards and Richardson [23] included 19 randomized controlled trials (RCTs) of 10 guided and 9 self-guided interventions for depression. The effect size found for the guided internet-based interventions was 0.78, and for the self-guided interventions the effect size was 0.36 (Cohen *d*). A study that directly compared guided and self-guided interventions for depression within an experimental comparison of the same program with or without therapeutic support and a control group found an effect size of 0.66 for the self-guided version and 1.14 for the guided version (outcome: Beck Depression Inventory-II or BDI-II), which showed that although the effects were smaller for self-guided than for guided, interventions without support were still effective [30]. A meta-analysis by Baumeister et al [31] also supports the superiority of guided over unguided interventions in terms of effectiveness; however, the extent of this superiority seems to be significantly smaller than that in most previous studies (especially in depression studies).

The advantage of self-guided internet-based interventions over guided interventions is that they provide increased access to treatment for those who need it, even for individuals who do not meet the full criteria of a disorder, and, at the same time, are affordable and conserve resources [21]. A recent meta-analysis used individualized participant data to estimate aggregated effect sizes in 13 RCTs on self-guided internet-based interventions for depression [29]. This type of meta-analysis is better able to identify the true effects while taking into account the variability of the studies (eg, degree of support, adherence to treatment, and setting). The meta-analysis found an effect size of 0.27 (Hedge *g*). Contrary to previous results showing that higher baseline symptoms predict a greater reduction of symptoms after the intervention period [32], baseline depressive symptoms did not moderate treatment outcome. Another important finding was that better adherence was associated with better treatment outcome.

### Objective

Self-guided internet-based interventions have several advantages over guided interventions and generally are effective in treating psychiatric symptoms. However, the question of which individuals benefit the most has not been investigated well enough. The aim of this study (NCT03795480) was to investigate the acceptance and effectiveness of a new self-guided internet-based intervention for depressive symptoms in a sample that had already received a similar intervention in the context of an earlier study [33]. In other words, we aimed to investigate the possible benefit of recurrent use of self-guided internet-based interventions. The intervention, called MOOD, was developed to provide individuals experiencing subjective depressive symptoms with low-threshold, self-directed, anonymous, and free access to an unguided internet-based intervention. Most of our participants had previously used a Web-based self-help program for depression in the framework of another trial [33]. The study thus enabled us to investigate whether people who had already received a similar therapy could still benefit from MOOD. We expected that participants who received access to MOOD would show a significant reduction of depressive symptoms (primary outcome: BDI-II) compared with the care-as-usual (CAU) control group. In addition, participants in the intervention group were expected to report a significant increase in self-esteem and quality of life after the intervention period, compared with the control group. Furthermore, willingness to change (as assessed with the University of Rhode Island Change Assessment (URICA) scale [34]) was expected to moderate treatment outcome. Another aim of the study was to examine possible moderators of treatment outcome.

## Methods

### Study Design

The study was a randomized controlled superiority trial with 2 conditions and parallel assignment (1:1). During the intervention period of 6 weeks, the intervention group received access to the internet-based self-help intervention MOOD, whereas the CAU group received access after completion of the post assessment. There were 2 assessment times, baseline and posttreatment (with an intervention period of 6 weeks). The study was approved by the local psychological ethics committee of the Center for Psychosocial Medicine of the University Medical Clinic Hamburg-Eppendorf, Germany (approval number: LEPEK-003). All participants gave Web-based informed consent before participating in the study. The study was conducted in accordance with the Declaration of Helsinki. The 2 assessments did not ask for any personal information except for an anonymous email address (instructions on how to create such an address were given); no names, telephone numbers, or addresses were asked for. Email addresses were kept in a handwritten list and were assigned to participant codes. The email addresses were stored in a safe. All other obtained data were anonymized and stored electronically on password-protected computers. If a participant requests the deletion of his or her data after completion of the study, this could be done if they provide the code word. The program MOOD ensures network security via secure sockets layer encryption. Messages that were sent within the internal message system of MOOD met the required data safety standard.

### Procedure

The study was conducted at the University Medical Center Hamburg-Eppendorf (Germany). At the 2 assessment times, baseline and posttreatment, data were obtained via an internet survey (Enterprise Feedback Suite survey from QuestBack Unipark). The baseline assessment obtained sociodemographic and psychopathological data (see subsection *Instruments*). After 6 weeks, all participants were invited to take part in the post assessment in which the same psychopathological data as before were assessed. Individuals in the treatment group were asked to provide a subjective evaluation of the internet-based self-help intervention. At the beginning of the post assessment, participants were asked to enter the same email address and individual code (numbers and letters) they had generated during the baseline assessment to ensure correct matching of pre and post data. Participants who completed the post assessment were rewarded with access to 2 mindfulness-based self-help manuals. These manuals contain a series of established relaxation and mindfulness exercises (eg, to increase acceptance and self-esteem) and were sent to the participants as PDF files.

### Sample size

The power analysis for calculating the sample size for an analysis of covariances (ANCOVA) was conducted using G*Power [35] and revealed a sample size of 128 to detect a medium effect of 0.25, with alpha=.05 and a power of .80, which was expected based on a meta-analysis of the effectiveness of internet-based cognitive behavioral therapy (CBT) for depression [23].

### Recruitment

The sample was recruited via an internal database of individuals with depression who had previously participated in studies conducted by the principle investigator’s unit and had given consent that they could be contacted for further studies. Most of the participants had previously participated in the Effectiveness of Internet-Based Depression Treatment study (EVIDENT) investigating the effectiveness of an internet-based self-help program for mild to moderate depressive symptoms [33]. Study invitations were sent via email providing information on the purpose and procedure of the study. In addition, invitations to the study were posted on online forums on depression and depression information websites. If participants were interested in taking part in the study, they were directed to a Web page from which the baseline assessment could be started. No financial compensation was offered.

### Eligibility Criteria

Participants could be included if they fulfilled the following inclusion criteria: subjective psychological distress with desire for treatment for depressive symptoms (there were no cutoff criteria for depressive symptoms at baseline), aged between 18 and 65 years, internet access, and sufficient command of the German language. Individuals with acute suicidality (assessed at baseline using item 9 on suicidal thoughts of the BDI-II, cutoff ≥2) and/or a self-reported lifetime diagnosis of schizophrenia or bipolar disorder were excluded from the study. Participants who were excluded because of acute suicidality were contacted and provided with help offers and telephone numbers that could be contacted in case of acute crisis. Participants with other psychiatric diagnoses were not excluded from the study. All participants were allowed to continue previously started treatments (psychotherapy or pharmacotherapy; *access to treatment*) and also changes in medication or psychotherapy were allowed during the participation.

### Randomization

Participants were randomly allocated into 1 of 2 conditions according to a randomization plan that was set up by the second author using the software Research Randomizer [36]. Block randomization was used to ensure balance between groups. As the study was conducted on the Web and the participants could actively enroll via Web-based registration, the allocation procedure differed from that in classical clinical trials, where allocation is performed by team members. On the basis of the date and time of completion of the baseline assessment, the participants were allocated to conditions following the randomization plan. The allocation rule was 1:1. Participants who were allocated into the intervention group received an email containing information on the program and a link to the login Web page of MOOD as well as individual login data in the form of a code and a password. Participants in the CAU group received an email with the information that they would receive access to MOOD after completion of the post assessment.

### Intervention

During the 6-week intervention period, the intervention group had access to MOOD, an internet-based self-help program targeting depressive symptoms. The program was developed by members of the neuropsychology working group of the University Medical Center Hamburg-Eppendorf and comprised 9 modules (see Table 1). The content of each module is based on CBT techniques and elements of the third wave of CBT. 

There is evidence that cognitive restructuring (modules ABC-protocol [A: activating event, B: belief, C: consequence] and modifying thoughts) and behavioral activation (module positive activities) are effective techniques in the treatment of depression [37,38]. In addition, the concept of mindfulness, which has received increasing attention in recent years, is addressed and practiced in a module labeled *mindfulness*. It has been shown that mindfulness has a beneficial effect on the outcome of psychotherapy [39-41]. Strengthening interpersonal skills and competences is strongly recommended within the treatment of depression [37,42] and, therefore, addressed in 1 module (social competence). It is also evident that depression is associated with sleep disturbances, which should, therefore, be targeted in treatment (module sleep) [43,44].

**Table 1:** Overview of MOOD modules.

Title	Description	Specific skills and exercises
Introduction	Presents the outline and goal of the program; discusses the interactions between thoughts, emotions, and behavior	Introduction into the principles of the program and strengthening of the treatment motivation; creation of list of values and needs according to which the user wants to live; emphasis is placed on the importance of interaction of thoughts, feelings, and behaviors
ABC protocol^a^	Highlights the importance of one’s beliefs in dealing with a specific situation; questions automatic beliefs about a situation and helps in developing new, more helpful beliefs	Introduction of the ABC protocol according to Ellis [45]; dysfunctional thought patterns (eg, all-or-nothing thinking and catastrophizing) are explained and converted to more helpful thoughts.
Positive activities	Shows how to integrate positive activities into one’s daily routine and achieve one’s goals	Presentation of lists of possible positive activities and exercises aimed at integrating positive activities into everyday life on a regular basis and planning them in a meaningful and realistic way; setting short- and long-term goals
Self-esteem	Makes the user aware of his or her own strengths and teaches strategies on how to improve his or her self-perception	The user is asked to identify personal sources of self-esteem and search for forgotten strength. Obstacles are addressed that could stand in the way of an increase of self-esteem (eg, unfair comparisons). Presentation of a list of concrete actions to increase self-esteem in everyday life (eg, joy diary)
Social competence	Presents ways to improve social competences to connect with other individuals and reach goals in social relations	Definition of social competence and presentation of characteristics of aggressive, unsafe, safe, and friendly behavior [46]
Mindfulness	Presents various mindfulness-based relaxation and attention exercises to increase mindfulness in daily life	Identification of signs of mindlessness; distinction between evaluations or judgements and observations to learn an inner attitude of conscious perception, neutrality, and acceptance; suggestions on how the user can distance himself from stressful feelings and obstructive thoughts; presentation of several classical mindfulness exercise (eg, body scan)—those are guided in audio files
Modifying thoughts	Uncovers depressive dysfunctional thoughts and explains methods to turn these into more realistic thoughts	Using ABC protocols to modify depressive dysfunctional thoughts; presentation of different possibilities to positively influence thoughts, such as making concrete statements, avoiding generalizations, changing perspectives, obtaining other opinions, and questioning situations
Sleep	Shows the importance of sleep quality and gives advice on how to improve sleep hygiene	Psychoeducation on the development and maintenance of sleep disorders; identification of characteristics of healthy sleep; creation of a personal list of tips for improving sleep behavior
Relapse prevention	Encourages paying attention to warning signals that might trigger depressive episodes and provides helpful coping strategies	Identification of possible triggers of relapse; presentation of suggestions on how these triggers can be avoided—the user is recommended to balance negative stress with positive activities and identify physical, emotional, cognitive, and behavioral warning signals for stress in oneself (according to a checklist from Kaluza [47]); development of an individual emergency plan

^a^A: activating event, B: belief, C: consequence.

All modules include interactive exercises, worksheets, pictures, graphics, videos, and audios that aim to incorporate the participant’s experiences and individual problems to increase the identification of the participant with the material and illustrate the content in an appealing way. The participants were free to choose the order of the modules and could work through the modules at their own speed. We have decided on a free choice of modules to give the participants experiences of autonomy and thus to minimize feelings of heteronomy (eg, being patronized; see [48] for a discussion of the role of motives in internet-based interventions). We recommended that they worked through 1 or 2 modules per week. The approximate time to finish a module ranged between 30 and 60 min. There was no direct guidance. However, the participants had the opportunity to contact a moderator in case of technical questions or problems via messaging within the program. This feature was optional, and the moderator did not actively contact the users on his or her own initiative. However, several reminders were sent via email to those participants who did not log into the program during the study. The participants were encouraged to have a look at the program and work with it. A short overview with summaries of the content of the modules was attached. The emails only served as a reminder to login to the program at least once; no therapeutic support was offered. Of the 55 participants (55/62, 89%) of the MOOD group who logged in to the program, 5 (5/55, 9%) used this feature. A total of 3 participants contacted the moderator several times—2 had 2 contacts (2/55, 4%), 1 had 3 contacts (1/55, 2%). Within the intervention period, none of the participants in the CAU group contacted the study staff.

### Instruments

Psychopathological self-rating questionnaires were assessed at the baseline and post assessments. The BDI-II [49] served as the primary outcome. Secondary outcomes included changes in self-esteem and quality of life as well as the participants’ subjective evaluation of MOOD.

#### Beck Depression Inventory-II

The BDI-II [49] was used to assess depressive symptom severity over the previous 2 weeks. The self-rating questionnaire comprises 21 items; for each item, the participant is asked to evaluate the severity of the symptom on a rating scale from 0 to 3, with higher scores indicating more severe depressive symptoms. An overall score of 0 to 13 indicates minimal depression, 14 to 19 indicates mild depression, 20 to 28 indicates moderate depression, and 29 to 63 suggests severe depression. The internal consistency of the BDI-II ranges from 0.79 to 0.90 [50].

#### Patient Health Questionnaire–9—Depression Module

Change in depressive symptoms was also assessed with the Patient Health Questionnaire–9 (PHQ-9) [51]. The PHQ-9 is a self-rating questionnaire that comprises 9 items on depression, which can be answered on a 4-point rating scale ranging from 0= *not at all* to 3= *nearly every day*. Sum scores can range from 0 to 27 with the following classifications: none or minimal (0-4), mild (5-9), moderate (10-14), and severe (15-27) depressive symptoms. Results of the questionnaire can assist in determining a diagnosis of MD according to Diagnostic and Statistical Manual of Mental Disorders, 4th Edition, criteria. Its internal consistency ranges from 0.86 to 0.89 [50].

#### Rosenberg Self-Esteem Scale

The Rosenberg Self-Esteem (RSE) scale [52] was used to assess self-esteem. The scale comprises 10 statements regarding self-esteem. Participants are instructed to rate how much they agree with the statements on a 4-point Likert scale from *strongly agree* to *strongly disagree*. Its internal consistency ranges from 0.77 to 0.88. In its original form, higher scores reflect less self-esteem; however, in our study we used a reversed rating scale such that higher scores reflect more self-esteem.

#### World Health Organization Quality of Life–Abbreviated Version

The WHO Quality of Life–abbreviated version (WHOQOL-BREF) assesses quality of life [53]. It is a short version of the WHOQOL-100, with 26 items. The questionnaire contains 4 different types of 5-point rating scales that ask the participant *how much*, *how complete*, *how often*, *how good*, or *how satisfied* he or she felt over the previous 2 weeks. The questionnaire has 4 subscales: physical health, psychological, social relations, and environment. The WHOQOL-BREF has an internal consistency of 0.70 [54].

#### University of Rhode Island Change Assessment

The URICA scale is a measure of willingness to change [34] and was used in the baseline survey. It comprises 32 items representing 4 phases of change: precontemplation, contemplation, action, and maintenance. In this study, a total of 9 items were used, selected from the subscales of precontemplation, contemplation, and action. The internal consistency is 0.83, and the reliability of the test-retest lies between 0.63 and 0.75. Answers could be given on a 5-point Likert scale ranging from 1 (strong disagreement) to 5 (strong agreement). In addition, a single item on expectation regarding the treatment outcome was added: “At present, how successful do you think the MOOD self-help program will be?” Here, answers could be given on a 9-point Likert scale ranging from 1 (*not successful at all*) to 9 (*very successful*).

#### Subjective Appraisal

The subjective appraisal of the intervention was assessed with questions we generated, as well as with adaptions of the Fragebogen zur Patientenzufriedenheit ZUF-8 (questionnaire to measure patient satisfaction [55]). The internal consistency of the ZUF-8 ranges between 0.87 and 0.93. Items could be answered on a 4-point Likert scale ranging from *totally disagree* to *totally agree*. Open questions gave participants the opportunity to provide feedback on the program.

### Statistical Analyses

Intention-to-treat (ITT), per-protocol (PP), and frequent user analyses were conducted using IBM Statistics 25 software. The ITT sample comprised all participants who participated in the baseline assessment, whereas the PP sample comprised those who completed both the baseline and the post assessments and used the intervention at least once during the intervention period. Those who used MOOD at least once a week were considered frequent users. According to the Consolidated Standards of Reporting Trials guidelines for reporting RCTs, both ITT and PP analyses should be reported [56]. Although reporting ITT analyses might be considered as the standard analysis for clinical trials, the PP analyses provide an estimate of the true efficacy, as it only includes participants who completed the study and showed (partial) adherence. Missing values in the ITT analysis were imputed using an expectation-maximizing algorithm with 200 imputations. For each measure, we conducted ANCOVA for all samples (ITT, PP, and frequent user) with pre-post differences as the within-group factor, condition as the between-group factor, and baseline scores as the covariate to account for regression toward the mean [57]. In addition, an exploratory moderation analysis was conducted for the PP sample to identify possible moderators that affected differential symptom improvement for the 2 conditions (outcome measure: BDI-II difference scores) using the SPSS macro PROCESS by Hayes [58].

## Results

### Baseline Characteristics

**Figure 1:**
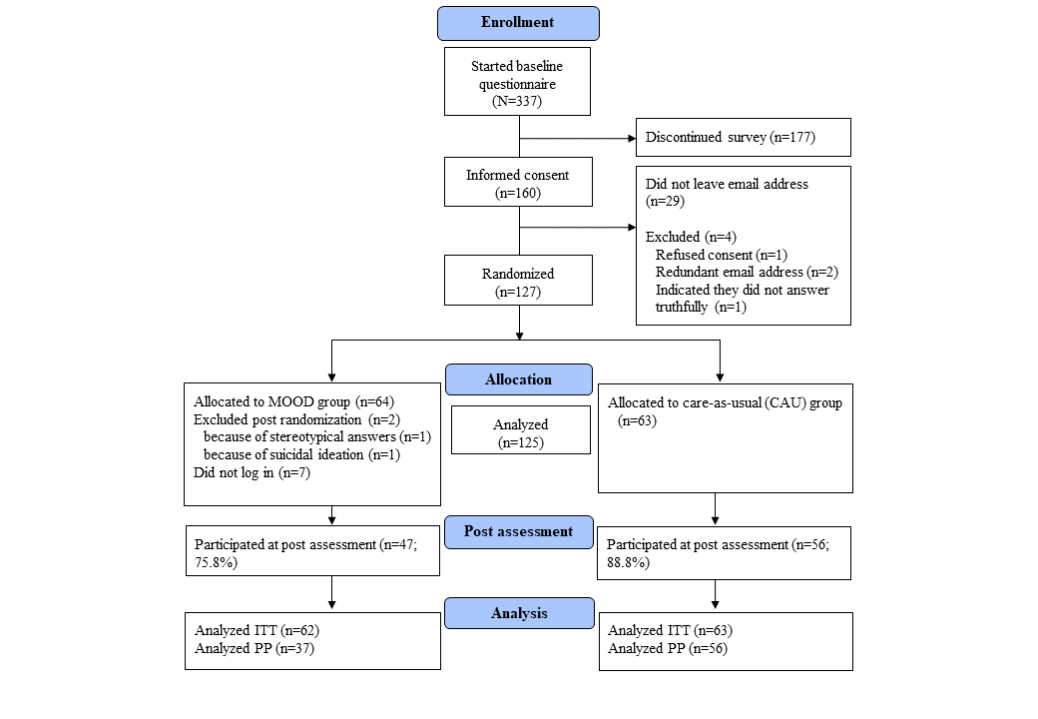
Consolidated Standards of Reporting Trials flow diagram. ITT: intention-to-treat; PP: per-protocol.

Table 2 shows the demographic and psychopathological data of the sample at baseline. The first participant was included on May 8, 2018, and the last post assessment took place on July 22, 2018. In total, 125 participants were included in the analyses. Of these, 63 were randomized to the CAU condition and 62 to the MOOD condition (see the study flowchart in Figure 1). Depression symptom severity was on average mild to moderate as measured by the BDI-II: 24/125 (19.2%) had minimal symptom severity (scores 0-13), 29/125 (23.2%) showed mild symptoms (scores 14-19), 39/125 (31.2%) had moderate symptoms (scores 20-28), and 33/125 (26.4%) had severe symptoms (scores 29-63). Of the sample, 49/125 (39.2%) currently were receiving outpatient psychotherapy and 5/125 (4.0%) were waiting for therapy.

**Table 2:** Demographic, psychopathological, and treatment variables along with respective statistical values (N=125).

Baseline characteristics			MOOD (n=62)	Care-as-usual (n=63)
**Demographic characteristics**				
	Male, n (%)		15 (24)	18 (29)
	Age (years), mean (SD)		44.02 (10.90)	48.02 (10.95)
	Level of school education (% A-level), n (%)		37 (60)	36 (57)
**Treatment variables**				
	Length of distress (years), mean (SD)		11.63 (7.97)	11.47 (7.91)
	**Medication, n (%)**			
		None	38 (61)	35 (56)
		Antidepressant	20 (32)	22 (35)
		Antidepressant and antipsychotics	4 (7)	6 (10)
	**Treatment status, n (%)**			
		None	27 (44)	28 (44)
		In treatment	24 (39)	25 (40)
		Waiting for therapy	2 (3)	3 (5)
	Treatment expectation^a^, mean (SD)		5.26 (1.55)	5.63 (1.42)
	**Number of previous courses of psychotherapy, n (%)**			
		0	4 (7)	9 (14)
		1-2	34 (55)	23 (37)
		>2	24 (39)	31 (49)
**Psychometric scales, mean (SD)**				
	BDI-II^b^ (depressive symptom severity)		22.54 (11.39)	22.79 (11.98)
	PHQ-9^c^ (depressive symptom severity)		10.74 (4.50)	10.52 (5.17)
	**WHOQOL-BREF^d^**			
		Global	48.39 (21.88)	49.80 (21.47)
		Physical health	55.59 (18.27)	57.31 (19.18)
		Psychological	43.55 (18.74)	45.90 (18.48)
		Social relationships	45.43 (20.56)	48.41 (20.62)
		Environmental	67.69 (16.47)	73.61 (16.01)
	RSE^e^ (self-esteem)		26.14 (6.96)	26.92 (6.92)

^a^1=*not at all successful* to 9=*very successful*.

^b^BDI-II: Beck Depression Inventory-II.

^c^PHQ-9: Patient Health Questionnaire–9.

^d^WHOQOL-BREF: World Health Organization Quality of Life–abbreviated version.

^e^RSE: Rosenberg Self-Esteem.

### Completion of Assessments and Treatment Adherence

The completion rate of the assessments was satisfactory; 103/125 (82.4%) completed the post assessment. There were no significant differences in completion of assessments between the intervention group and the CAU group (χ²_1,125_=3.7; *P*=.06). Completers and noncompleters were not significantly different on any baseline demographic characteristic except for 2 items regarding the presence of a diagnosis of a depressive disorder and substance or alcohol dependency. Specifically, of those who did not complete the post assessment, 6/22 (27%) did not have a self-reported diagnosis of depression, whereas of those who completed the post assessment, 9/103 (8.7%) did not have a self-reported diagnosis of depression (χ²_1,125_=5.9; *P*=.02). Relatively more participants, 3/22 (14%), who did not complete the post assessment indicated having a diagnosis of a substance or alcohol dependency compared with those who completed the study and had such a diagnosis (3/103, 2.9%; χ²_1,125_=4.6; *P*=.03).

Although the completion rate of the assessments was satisfactory, the usage of the intervention was rather low. Only 24/62 (39%) logged into the program at least once a week (frequent users). Of those who logged into the program, the participants completed an average of 2.90 (SD 3.13) modules. The mean time (in minutes) the users engaged with the program was 117.36 min (SD 209.05). No significant correlation could be found between duration of use and the number of completed modules with symptom improvement after the intervention (*P*>.05).

### Primary Outcome

Tables 3 and 4 shows the group differences across time of each measure for the PP sample, frequent user sample (those who logged into the program at least once a week), and ITT sample. For the primary outcome (score on the BDI-II), the results of the ANCOVA analysis did not reach statistical significance, neither for ITT nor PP or frequent user sample. Results of the paired sample *t* tests showed a significant decline of depressive symptoms from pre to post for the intervention (*t*_46_=2.05; *P*=.05) and CAU (*t*_55_=2.96; *P*=.004) groups.

**Table 3:** Group differences across time; means, standard deviations, effect sizes (Cohen *d*) and 95% CIs of completer sample (within-group differences are denoted via superscripts).

Measurements		MOOD			Care-as-usual		
		Pre (n=62), mean (SD)	Post (n=47), mean (SD)	Cohen *d* (95% CI)	Pre (n=63), mean (SD)	Post (n=56), mean (SD)	Cohen *d* (95% CI)
BDI-II^a^		22.54 (11.39)	20.36 (14.70)	−0.17 (−0.55 to 0.21)^b^	22.79 (11.99)	18.68 (12.79)	−0.35 (−0.71 to 0.02)^c^
PHQ-9^d^		10.74 (4.50)	10.60 (6.23)	−0.03 (−0.41 to 0.35)	10.52 (5.17)	9.11 (5.64)	−0.26 (−0.62 to 0.01)^b^
**WHOQOL-BREF^e^**							
	Global	48.39 (21.88)	50.00 (19.85)	0.08 (−0.30 to 0.46)	49.80 (21.47)	55.13 (22.71)	0.24 (−0.12 to 0.60)^b^
	Physical health	55.59 (18.28)	57.22 (20.33)	−0.09 (−0.29 to 0.46)	57.31 (19.18)	60.33 (19.89)	0.16 (−0.21 to 0.52)
	Psychological	43.55 (18.74)	46.72 (20.43)	−0.16 (−0.22 to 0.54)^f^	45.90 (18.48)	49.70 (19.54)	0.20 (−0.16 to 0.56)^b^
	Social relationships	45.43 (20.56)	48.40 (23.03)	−0.14 (−0.24 to 0.52)	48.41 (20.62)	50.30 (20.59)	0.09 (−0.27 to 0.45)
	Environment	67.69 (16.47)	70.01 (14.09)	−0.15 (−0.23 to 0.53)^b^	73.61 (16.01)	76.28 (15.17)	0.17 (−0.19 to 0.53)^b^
RSE^g^		26.15 (6.96)	27.34 (7.67)	−0.16 (−0.22 to 0.54)^c^	26.92 (6.12)	28.68 (6.74)	0.27 (−0.09 to 0.64)^c^

^a^BDI-II: Beck Depression Inventory-II.

^b^*P*≤.05.

^c^*P*≤.005.

^d^PHQ-9: Patient Health Questionnaire–9.

^e^WHOQOL-BREF: World Health Organization Quality of Life–abbreviated version.

^f^*P*≤.01

^g^RSE: Rosenberg Self-Esteem.

**Table 4:** Analysis of covariances with respective baseline values as covariates.

Measurements		Per protocol sample (n=93)		*P* value	Frequent user sample (n=80)		*P* value	Intention to treat (n=125)		*P* value
		*F*_1,90_	η_p_^2^		*F*_1,77_	η_p_^2^		*F*_1,122_	η_p_^2^	
BDI-II^a^		0.24	0.003	.63	0.24	0.003	.63	0.94	0.008	.34
PHQ-9^b^		0.03	0.000	.86	0.00	0.000	.97	2.41	0.019	.12
**WHOQOL-BREF^**c**^**										
	Global	0.10	0.001	.75	0.03	0.000	.87	1.72	0.014	.19
	Physical health	0.66	0.007	.42	0.04	0.001	.84	0.37	0.003	.55
	Psychological	0.57	0.006	.45	1.75	0.022	.19	0.11	0.001	.75
	Social relationships	0.69	0.008	.41	0.33	0.004	.57	0.10	0.001	.75
	Environment	0.01	0.000	.95	0.27	0.003	.61	0.92	0.008	.34
RSE^d^		0.879	0.010	.35	0.51	0.007	.48	0.44	0.004	.51

^a^BDI-II: Beck Depression Inventory-II.

^b^PHQ-9: Patient Health Questionnaire–9.

^c^WHOQOL-BREF: World Health Organization Quality of Life–abbreviated version.

^d^RSE: Rosenberg Self-Esteem.

### Secondary Outcomes

Results of paired sample *t* tests indicate a significant increase in scores on the self-esteem scale (RSE) for the intervention (*t*_46_=2.97; *P*=.005) and CAU (*t*_55_=3.04; *P*=.004) groups. There was no significant improvement across time in scores on the RSE as analyzed using an ANCOVA with baseline score as covariate (see Table 4). This is applicable to all samples analyzed (ITT, PP, and frequent user). Quality of life was assessed with the WHOQOL-BREF. Although there was a significant increase in the global score of the CAU control group (*t*_55_=2.46; *P*=.02), there was no significant increase in the intervention group (*t*_46_=1.09; *P*=.28). Paired sample *t* tests for the subscales of the WHOQOL-BREF (physical health, psychological, social relationships, and environment) found a significant increase on the WHOQOL-BREF psychological (MOOD: *t*_46_=−2.86; *P*=.006 and CAU: *t*_55_=−2.50; *P*=.02) and environmental (MOOD: *t*_46_=−2.08; *P*=.04 and CAU: *t*_55_=−2.27; *P*=.03) scales for both groups. The ANCOVA analysis did not find a significant difference across groups for the WHOQOL-BREF and its subscales.

### Moderation Analysis

**Figure 2:**
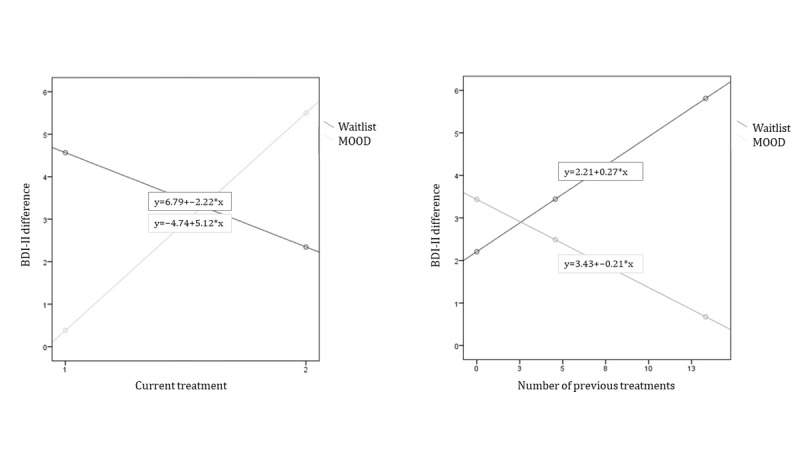
Interaction effects of current treatment (left), number of prior treatment (right) and group allocation. The graph on the left presents the effects of current treatment (1=yes, 2=no) on symptom reduction. The graph on the right depicts how experience with psychotherapy (number of prior treatments) is related to depressive symptomatology (outcome: reduction on BDI-II). BDI-II: Beck Depression Inventory-II.

The results for the interaction effect of the exploratory moderation analyses are summarized in Table 5. Subsequent analyses showed that participants in the intervention group who had less experience with psychotherapy showed a greater improved outcome on depression (BDI-II) compared with the CAU group (*P*=.03). In addition, moderation analyses showed results that bordered on significance (*P*=.05), suggesting that individuals in the intervention group who were currently receiving no other treatment had a better outcome compared with the CAU group (see Figure 2 and Table 5). Results indicate that willingness to change did not moderate treatment outcome (*P*>.05).

**Table 5:** Moderators for improvement in depression (Beck Depression Inventory-II difference score, means are centered).

Outcome parameter	Beta	SE	*t*	*P* value	95% CI
Number of past courses of psychotherapy	−.478	0.219	−2.178	.03	−0.913 to −0.042
Current treatment^a^	7.340	3.711	1.978	.05	−0.035 to 14.714

^a^Possible answers were no therapy, in-patient treatment, out-patient treatment, treatment with licensed psychotherapist, treatment at day hospital, waiting for therapy to begin, and planning on starting therapy.

### Subjective Appraisal

In Tables 6 and 7, subjective appraisals of MOOD are displayed. Overall, MOOD was positively evaluated. Of those who used the intervention, 78% (29/37) rated MOOD as suitable for self-help, 73% (27/37) considered it an applicable supplement to psychotherapy, and 54% (20/37) rated the program as helpful. Furthermore, 81% (30/37) rated the quality of the program as excellent to good, 76% (28/37) would recommend it to a friend with similar problems, and 68% (25/37) would use the program again. However, only 22% (8/37) indicated that they were able to use MOOD regularly, 46% (17/37) had to force themselves to use the program, and only 30% (11/37) stated that their depressive symptoms decreased through using MOOD. In addition, 60% (18/30) rated MOOD to be equally good or better than the internet-based intervention they had previously used, and 17% (5/30) considered MOOD inferior.

**Table 6:** Subjective appraisal of MOOD (scores: 1=not at all, 2=a little, 3=a lot, and 4=absolutely).

Item	MOOD condition (n=37), mean (SD)	Positive (*a lot* and *absolutely*) appraisal, n (%)
I think the MOOD program is good for self-help and self-guidance.	2.95 (0.85)	29 (78)
I think the contents of the program were understandable.	1.97 (0.93)	35 (95)
I think the program was helpful.	2.54 (1.02)	20 (54)
I was able to use the program on a regular basis during the past 6 weeks.	1.86 (0.95)	8 (22)
I had to force myself to use the program.	2.46 (1.17)	17 (46)
My depressive symptoms decreased because of the use of the program.	1.97 (0.93)	11 (30)
I consider the program to be applicable as a supplement to psychotherapy.	2.97 (0.87)	27 (73)
The program is not applicable to my depressive symptoms.	1.76 (1.07)	9 (24)

**Table 7:** Subjective appraisal of MOOD (adapted from a German questionnaire on patient satisfaction, ZUF-8 [59]).

Item	MOOD condition (n=37), mean (SD)	Positive appraisal, n (%)
How do you rate the quality of the program? (excellent, good vs okay, not good)	2.30 (1.05)	30 (81)
Did you receive the type of treatment you expected to receive? (absolutely, a lot vs a little, not at all)	3.16 (0.90)	25 (68)
To what extent did the program help you cope with your problems? (absolutely, a lot vs a little, not at all)	2.76 (1.14)	19 (51)
Would you recommend the program to a friend with similar symptoms? (yes, probably yes vs probably not, no)	3.32 (0.97)	28 (76)
How happy are you about the extent of the help you have received through using the program? (very satisfied, mostly satisfied vs somewhat dissatisfied, dissatisfied)	3.16 (1.07)	21 (57)
Did the program help you cope with your problems more successfully? (absolutely, a lot vs a little, not at all)	2.65 (1.36)	21 (57)
How satisfied are you with the program in general? (very satisfied, mostly satisfied vs somewhat unsatisfied, unsatisfied)	2.57 (1.28)	25 (68)
Would you use the program again? (Yes, probably yes vs probably not, no)	3.35 (1.11)	25 (68)

## Discussion

### Principal Findings

The treatment gap in the treatment of depressive disorders is a significant issue [8] and might be narrowed by providing effective internet-based interventions. Self-guided internet-based interventions can be especially useful because therapists are a scarce resource. Furthermore, depressive episodes tend to reoccur [60], but treatment barriers are stable, which means that affected individuals are repeatedly confronted with the problem of the undersupply of therapists. In our study, we aimed to investigate whether the use of a new self-guided internet-based intervention called MOOD over a period of 6 weeks would lead to a significant reduction in depressive symptoms compared with a CAU group (that got access to MOOD after completion of the post assessment) in a sample of individuals who had previously and/or currently received therapy (most of the participants had also already received a different internet-based intervention).

In our analyses, we found no significant difference across groups in any outcome variable (for ITT, PP, and frequent users). Contrary to our hypothesis, the group that received MOOD over the intervention period did not significantly improve in depressive symptoms compared with the CAU group. Both groups significantly improved over time and, interestingly, the difference here was more pronounced for the CAU group for the primary outcome, which was the BDI-II. Both groups significantly improved over time in levels of self-esteem and quality of life, but this effect again was not different across groups. These findings are different from previous trials investigating the effectiveness of self-guided internet-based interventions. Here, it was found that self-guided internet-based interventions were effective in treating depressive symptoms at a small to medium effect size [21,23]. Certainly, it is possible that this difference between ours and previous results might stem from a publication bias. Furthermore, self-guided internet-based interventions were found to be more effective in individuals currently not seeing a psychotherapist [33,61]. Results of this study support this finding with a *P* value of .05, which indicates that individuals in the intervention group who benefited more from the program were currently not receiving other psychotherapy. However, this result was not statistically significant. The number of participants who were in treatment while using MOOD amounted to 39%, and these individuals had a smaller symptom reduction compared with those who were not in treatment. This result indicates that internet-based interventions appear to be more useful to individuals who are currently not receiving psychotherapy. More than 70% of participants in the intervention group considered MOOD to be a suitable supplement to psychotherapy. However, this is more likely to be the case if the combination of internet-based interventions and psychotherapy is addressed in therapy, and the integration is actively promoted, as in blended therapy [62,63,64].

Another interesting finding was that the participants’ past experience with psychotherapy had a significant effect on treatment outcome. Here, results indicated that individuals with less experience benefited more than those with more experience. Our sample mainly comprised individuals with a relatively long history of depression; most of them had undergone multiple courses of psychotherapy in the past. Only 10.4% of the overall sample had not previously received psychotherapy. Also, 82.9% had previously used a comparable self-guided internet-based intervention within the framework of another trial [33]. Our results support the assumption that individuals who have already used a very similar internet-based intervention and have a high level of psychotherapeutic experience may not benefit from another self-guided Web-based intervention. Perhaps they should receive personalized support, such as a guided internet-based intervention. It is conceivable that individuals who have suffered from depression for a long time and who already have had significant treatment experience will not learn much that is unknown or new to them in a subsequent intervention. Another explanation might be that individuals who have not benefited from conventional psychotherapy and, therefore, have continued to look for other forms of treatment also will not benefit from internet-based treatment. The fact that people with a long history of depression usually suffer from residual symptoms that are difficult to treat is a well-known prognostic factor and therefore is presumably another reason for the nonsignificant group differences we found in our sample [65,66]. Another important point is that patient’s expectations of the outcome of therapy is considered a key determinant of the actual therapy outcome [67,68]. Many patients with a long history of a mental disorder and extensive therapy experience are likely to have lower expectations and hopes regarding treatment outcome. This naturally applies to other forms of therapy too and is not confined to internet-based interventions. However, it could be a further reason for the lack of treatment effects found in our study. Despite this, based on the results presented above, we come to the conclusion that unguided internet-based interventions should mainly serve as a first step in treatment and are not suitable for individuals with recurrent or residual symptoms. Our findings, therefore, support stepped care approaches, in which self-guided internet-based interventions are used in early phases of illness and in patients without a chronification of symptoms [69-71]. The key idea behind these stepped models is that patients should first be treated at the lowest level (to avoid *psychiatrization* and self-stigma) and only proceed to more advanced care when symptoms are severe or do not improve with (guided) self-help. It has been found that stepped care approaches can be effective and resource-saving in the treatment of common mental disorders, such as depression [72]. Another potential implication is that programs for individuals with therapeutic experience have to be more elaborated to be effective. For instance, they could consider motives of participants [48] or be personalized in other ways.

### Strengths and Limitations

To participate in the study, neither a (verified) diagnosis of depression nor a minimum severity of depression symptoms was necessary, which resulted in heterogeneity of depression levels. Therefore, on the one hand, a wider range of individuals with desire for treatment for depression was reached, regardless of whether they met the criteria of a diagnosis or not. On the other hand, it has been found that samples of severely depressed participants benefit more from low-intensity psychological interventions than samples of mildly depressed participants [32], which might be because those with severe depression have more room to improve. This finding, however, contrasts with results of Karyotaki et al [21] who found that self-guided internet-based interventions are effective regardless of symptom severity. Interestingly, within another study by Karyotaki et al [73], it was found that individuals with more severe baseline symptoms were more likely to improve than individuals with less severe baseline symptoms after treatment with guided internet-based interventions, which means that the findings are still ambiguous in this respect. Apart from this, broad inclusion criteria may result in a type I error and, thereby, lead to an underestimation of the intervention’s treatment potential. Another limitation might be the sample size in our study. Our power analysis was based on a medium effect, and we did not consider expected dropout in our sample size calculation, which could have led to an overestimation, as Karyotaki et al [21] only found a small effect (g=0.27) for self-guided interventions. To detect such a small effect, a larger sample would have been necessary.

A strength of the study is the high completion rate of the assessments—only 17.6% did not participate or complete the post assessment. Despite the high completion rate, the treatment adherence was rather low; only 39% were able to use the program on a regular basis (at least once a week), and the average time of program usage was 117.36 min. Contrary to the results of other studies, there was no significant correlation between duration of use and therapy outcome [21,74]. Interestingly, the percentage of participants who self-reported a regular use was minor (22%) compared with the percentage of the frequent users as defined by us. It is conceivable that depressive users themselves define much higher standards for regular use. If they do not meet these demands, this leads to disappointment and negative feelings. This point can be discussed in the context of depression-specific negative cognitive distortions, namely, (dysfunctional) perfectionism and *all-or-nothing thinking* [75,76]. Individuals with depression, who have these thought distortions, often think that an expectation task is either fulfilled perfectly (100%) or not at all. In our case, this could mean that the participants rated themselves or their use of the program worse than they actually did because of their depressive way of thinking. Strategies that improve adherence in self-guided internet-based interventions (such as sending reminders) have been found to have a positive effect on treatment outcome [77]. However, in our study, we did send frequent reminders to participants to use the intervention. Finally, as the study had limited financial resources, follow-up assessments were not possible. For this reason, no conclusion can be drawn about long-term effects.

### Conclusions

The efficacy of unguided internet-based interventions has been proven in various trials. However, it is unclear whether internet-based interventions are beneficial for individuals with a long history of depression and greater experience with psychotherapy and for those who have already undergone a different unguided Web-based intervention. With this sample, we could not verify the efficacy of the new self-help internet-based intervention MOOD; however, 2 interesting findings with implications for the future were identified. Greater experience with psychotherapy and (at trend level) current treatment reduced the effects of the intervention on depressive symptoms. We conclude that unguided internet-based interventions might be appropriate as a first step in treatment but not for individuals who already have much experience with psychotherapy or internet-based interventions.

## Appendix

Multimedia Appendix 1 CONSORT‐EHEALTH checklist (V 1.6.1).

## Abbreviations

ABC (-protocol): activating event, beliefs, consequence
ANCOVA: analysis of covariances
BDI-II: Beck Depression Inventory-II
CAU: care-as-usual
CBT: cognitive behavioral therapy
ITT: intention-to-treat
MD: major depression
PHQ-9: Patient Health Questionnaire–9
PP: per-protocol
RCT: randomized controlled trial
RSE: Rosenberg Self-Esteem
URICA: University of Rhode Island Change Assessment
WHO: World Health Organization
WHOQOL-BREF: World Health Organization Quality of Life–abbreviated version
ZUF-8: Fragebogen zur Patientenzufriedenheit (patient satisfaction questionnaire)
