# Supplementary material for: Diminishing Effects After Recurrent Use of Self-Guided Internet-Based Interventions in Depression: Randomized Controlled Trial
Source: J Med Internet Res. 2019 Oct 2;21(10):e14240. doi: 10.2196/14240 (PMC6777284; doi:10.2196/14240)
Supplement: Multimedia Appendix 1 [file jmir_v21i10e14240_app1.pdf]

CONSORT-EHEALTH Checklist V1.6.2 Report	Manuscript Number	14240

(based on CONSORT-EHEALTH V1.6), available at [http://tinyurl.com/consort-ehealth-v1-6].

Date completed

9/9/2019 9:33:47

by

Lara Bücker

Recurrent Use of Self-Guided Internet-Based Interventions for Depression: Randomized Controlled Trial

TITLE

1a-i) Identify the mode of delivery in the title

"Recurrent Use of Self-Guided Internet-Based Interventions for Depression"

1a-ii) Non-web-based components or important co-interventions in title

This feature does not appear in the title as it is not of huge importance. However, unter the subsection "intervention" we write: "Several reminders were sent via email to those participants who did not log into the program during the study.The participants were encouraged to have a look at the program and work with it. A short overview with summaries of the content of the modules was attached."

1a-iii) Primary condition or target group in the title

"Recurrent Use of Self-Guided Internet-Based Interventions for Depression"

ABSTRACT

1b-i) Key features/functionalities/components of the intervention and comparator in the METHODS section of the ABSTRACT

In the abstract, we only write: "We aimed to investigate whether the use of a new self-guided internet-based intervention (MOOD) (...)" and did not specify the components of the intervention, as the abstract was intendet to be short. Under the subsection "intervention" the internet-based intervention is described in detail and a Table with all distinct modules, exercises, theories etc. is provided.

1b-ii) Level of human involvement in the METHODS section of the ABSTRACT

In the abstract, we write "We aimed to investigate whether the use of a new self-guided internet-based intervention (MOOD) (...)" and thus point out that the intervention is self-guided, which in my opinion is sufficient.

1b-iii) Open vs. closed, web-based (self-assessment) vs. face-to-face assessments in the METHODS section of the ABSTRACT

In order not to exceed the number of words in the abstract, this information has been omitted here. In the section "Randomization" we write: "As the study was conducted on the Web and the participants could actively enroll via Web-based registration, the   allocation procedure differed from that in classical clinical trials, where allocation is performed by team members." In the section "Procedure" we write: "At the 2 assessment times, baseline and posttreatment, data were obtained via an internet survey (Enterprise Feedback Suite survey from QuestBackUnipark)."

1b-iv) RESULTS section in abstract must contain use data

In the abstract, we provdie the total number of participants randomized into the two conditions. "A total of 125 individuals were randomized to the intervention condition (MOOD) and received access to the intervention for a period of 6 weeks or a CAU group."

1b-v) CONCLUSIONS/DISCUSSION in abstract for negative trials

"Self-guided internet-based interventions are deemed a suitable first-step approach to the treatment of depression. However, our results indicate that they are more efficacious in those with less psychotherapy experience."

INTRODUCTION

2a-i) Problem and the type of system/solution

"Internet-based interventions can help overcome the supply and demand barriers of conventional face-to-face treatment. Internet-based treatments could reach people who fear stigmatization, are widely accessible (especially for those living in rural areas or those with physical barriers), and provide a high level of privacy [14,15]. Furthermore, the individuals themselves can decide when and where they want to use the intervention. In addition, these interventions are usually available at low cost and, thereby, are affordable for individuals with limited financial resources" (...) "The advantage of self-guided internet-based interventions over guided interventions is that they provide increased access to treatment for those who need it, even for individuals who do not meet the full criteria of a disorder, and, at the same time, are affordable and conserve resources "

2a-ii) Scientific background, rationale: What is known about the (type of) system

"A recent meta-analysis used individualized participant data to estimate aggregated effect sizes in 13 RCTs on self-guided internet-based interventions for depression [29]. (...)The meta-analysis found an effect size of 0.27. " (...) "Self-guided internet-based interventions have several advantages over guided interventions and generally are effective in treating psychiatric symptoms. However, the question of which individuals benefit the most has not been investigated well enough. The aim of this study (NCT03795480) was to investigate the acceptance and effectiveness of a new self-guided internet-based intervention for depressive symptoms in a sample that had already received a similar intervention in the context of an earlier study."

Does your paper address CONSORT subitem 2b?

"The study thus enabled us to investigate whether people who had already received a similar therapy could still benefit from MOOD. We expected that participants who received access to MOOD would show a significant reduction of depressive symptoms (primary outcome: BDI-II) compared with the care-as-usual (CAU) control group. In addition, participants in the intervention group were expected to report a significant increase in self-esteem and quality of life after the intervention period, compared with the control group. Furthermore, willingness to change (as assessed with the University of Rhode Island Change Assessment (URICA) scale [34]) was expected to moderate treatment outcome. Another aim of the study was to examine possible moderators of treatment outcome."

METHODS

3a) CONSORT: Description of trial design (such as parallel, factorial) including allocation ratio

"The study was a randomized controlled superiority trial with 2 conditions and parallel assignment (1:1)."

3b) CONSORT: Important changes to methods after trial commencement (such as eligibility criteria), with reasons

Not applicable as no changes were made.

3b-i) Bug fixes, Downtimes, Content Changes

4a) CONSORT: Eligibility criteria for participants

"Eligibility Criteria

Participants could be included if they fulfilled the following inclusion criteria: subjective psychological distress with desire for treatment for depressive symptoms (there were no cutoff criteria for depressive symptoms at baseline), aged between 18 and 65 years, internet access, and sufficient command of the German language. Individuals with acute suicidality (assessed at baseline using item 9 on suicidal thoughts of the BDI-II, cutoff ≥2) and/or a self-reported lifetime diagnosis of schizophrenia or bipolar disorder were excluded from the study. Participants who were excluded because of acute suicidality were contacted and provided with help offers and telephone numbers that could be contacted in case of acute crisis. Participants with other psychiatric diagnoses were not excluded from the study. All participants were allowed to continue previously started treatments (psychotherapy or pharmacotherapy; access to treatment) and also changes in medication or psychotherapy were allowed during the participation."

4a-i) Computer / Internet literacy

4a-ii) Open vs. closed, web-based vs. face-to-face assessments:

"The sample was recruited via an internal database of individuals with depression who had previously participated in studies conducted by the principle investigator’s unit and had given consent that they could be contacted for further studies. Most of the participants had previously participated in the Effectiveness of Internet-Based Depression Treatment study investigating the effectiveness of an internet-based self-help program for mild to moderate depressive symptoms [33]. Study invitations were sent via email providing information on the purpose and procedure of the study. In addition, invitations to the study were posted on online forums on depression and depression information websites. If participants were interested in taking part in the study, they were directed to a Web page from which the baseline assessment could be started." (...) "As the study was conducted on the Web and the participants could actively enroll via Web-based registration, the allocation procedure differed from that in classical clinical trials, where allocation is performed by team members. On the basis of the date and time of completion of the baseline assessment, the participants were allocated to conditions following the randomization plan."

4a-iii) Information giving during recruitment

4b) CONSORT: Settings and locations where the data were collected

"The study was conducted at the University Medical Center Hamburg-Eppendorf (Germany)."

4b-i) Report if outcomes were (self-)assessed through online questionnaires

"At the 2 assessment times, baseline and posttreatment, data were obtained via an internet survey (Enterprise Feedback Suite survey from QuestBack Unipark). "

4b-ii) Report how institutional affiliations are displayed

5) CONSORT: Describe the interventions for each group with sufficient details to allow replication, including how and when they were actually administered

5-i) Mention names, credential, affiliations of the developers, sponsors, and owners

**5-ii) Describe the history/development process**		
**5-iii) Revisions and updating**		
**5-iv) Quality assurance methods**		
**5-v) Ensure replicability by publishing the source code, and/or providing screenshots/screen-capture video, and/or providing flowcharts of the algorithms used**		
**5-vi) Digital preservation**		
**5-vii) Access**		
"Participants who were allocated into the intervention group received an email containing information on the program and a link to the login Web page of MOOD as well as individual login data in the form of a code and a password. Participants in the CAU group received an email with the information that they would receive access to MOOD after completion of the post assessment."		
**5-viii) Mode of delivery, features/functionalities/components of the intervention and comparator, and the theoretical framework**		
"The program was developed by members of the neuropsychology working group of the University Medical Center Hamburg-Eppendorf and comprised 9 modules (see Table 1). The content of each module is based on CBT techniques and elements of the third wave of CBT. There is evidence that cognitive restructuring (modules ABC-protocol [A: activating event, B: belief, C: consequence] and modifying thoughts) and behavioral activation (module positive activities) are effective techniques in the treatment of depression [37,38]. In addition, the concept of mindfulness, which has received increasing attention in recent years, is addressed and practiced in a module labeled mindfulness. It has been shown that mindfulness has a beneficial effect on the outcome of psychotherapy [39-41]. Strengthening interpersonal skills and competences is strongly recommended within the treatment of depression [37,42] and, therefore, addressed in 1 module (social competence). It is also evident that depression is associated with sleep disturbances, which should, therefore, be targeted in treatment (module sleep) [43,44]. All modules include interactive exercises, worksheets, pictures, graphics, videos, and audios that aim to incorporate the participant's experiences and individual problems to increase the identification of the participant with the material and illustrate the content in an appealing way. The participants were free to choose the order of the modules and could work through the modules at their own speed. We have decided on a free choice of modules to give the participants experiences of autonomy and thus to minimize feelings of heteronomy (eg, being patronized; see [48] for a discussion of the role of motives in internet-based interventions). We recommended that they worked through 1 or 2 modules per week. The approximate time to finish a module ranged between 30 and 60 min. There was no direct guidance. However, the participants had the opportunity to contact a moderator in case of technical questions or problems via messaging within the program. This feature was optional, and the moderator did not actively contact the users on his or her own initiative. However, several reminders were sent via email to those participants who did not log into the program during the study. The participants were encouraged to have a look at the program and work with it. A short overview with summaries of the content of the modules was attached."		
**5-ix) Describe use parameters**		
**5-x) Clarify the level of human involvement**		
**5-xi) Report any prompts/reminders used**		
"The participants were encouraged to have a look at the program and work with it. A short overview with summaries of the content of the modules was attached."		
**5-xii) Describe any co-interventions (incl. training/support)**		
Not applicable as there were no co-interventions.		
**6a) CONSORT: Completely defined pre-specified primary and secondary outcome measures, including how and when they were assessed**		
"Psychopathological self-rating questionnaires were assessed at the baseline and post assessments. The BDI-II [49] served as the primary outcome. Secondary outcomes included changes in self-esteem and quality of life as well as the participants' subjective evaluation of MOOD.<br>Beck Depression Inventory-II<br><br>The BDI-II [49] was used to assess depressive symptom severity over the previous 2 weeks. The self-rating questionnaire comprises 21 items; for each item, the participant is asked to evaluate the severity of the symptom on a rating scale from 0 to 3, with higher scores indicating more severe depressive symptoms. An overall score of 0 to 13 indicates minimal depression, 14 to 19 indicates mild depression, 20 to 28 indicates moderate depression, and 29 to 63 suggests severe depression. The internal consistency of the BDI-II ranges from 0.79 to 0.90 [50].<br>Patient Health Questionnaire–9—Depression Module<br><br>Change in depressive symptoms was also assessed with the Patient Health Questionnaire–9 (PHQ-9) [51]. The PHQ-9 is a self-rating questionnaire that comprises 9 items on depression, which can be answered on a 4-point rating scale ranging from 0= not at all to 3= nearly every day. Sum scores can range from 0 to 27 with the following classifications: none or minimal (0-4), mild (5-9), moderate (10-14), and severe (15-27) depressive symptoms. Results of the questionnaire can assist in determining a diagnosis of MD according to Diagnostic and Statistical Manual of Mental Disorders, 4th Edition, criteria. Its internal consistency ranges from 0.86 to 0.89 [50].<br>Rosenberg Self-Esteem Scale<br><br>The Rosenberg Self-Esteem (RSE) scale [52] was used to assess self-esteem. The scale comprises 10 statements regarding self-esteem. Participants are instructed to rate how much they agree with the statements on a 4-point Likert scale from strongly agree to strongly disagree. Its internal consistency ranges from 0.77 to 0.88. In its original form, higher scores reflect less self-esteem; however, in our study we used a reversed rating scale such that higher scores reflect more self-esteem.<br>World Health Organization Quality of Life–Abbreviated Version<br><br>The WHO Quality of Life–abbreviated version (WHOQOL-BREF) assesses quality of life [53]. It is a short version of the WHOQOL-100, with 26 items. The questionnaire contains 4 different types of 5-point rating scales that ask the participant how much, how complete, how often, how good, or how satisfied he or she felt over the previous 2 weeks. The questionnaire has 4 subscales: physical health, psychological, social relations, and environment. The WHOQOL-BREF has an internal consistency of 0.70 [54].<br>University of Rhode Island Change Assessment<br><br>The URICA scale is a measure of willingness to change [34] and was used in the baseline survey. It comprises 32 items representing 4 phases of change: precontemplation, contemplation, action, and maintenance. In this study, a total of 9 items were used, selected from the subscales of precontemplation, contemplation, and action. The internal consistency is 0.83, and the reliability of the test-retest lies between 0.63 and 0.75. Answers could be given on a 5-point Likert scale ranging from 1 (strong disagreement) to 5 (strong agreement). In addition, a single item on expectation regarding the treatment outcome was added: "At present, how successful do you think the MOOD self-help program will be?" Here, answers could be given on a 9-point Likert scale ranging from 1 (not successful at all) to 9 (very successful).<br>Subjective Appraisal<br><br>The subjective appraisal of the intervention was assessed with questions we generated, as well as with adaptions of the Fragebogen zur Patientenzufriedenheit ZUF-8 (questionnaire to measure patient satisfaction [55]). The internal consistency of the ZUF-8 ranges between 0.87 and 0.93. Items could be answered on a 4-point Likert scale ranging from totally disagree to totally agree. Open questions gave participants the opportunity to provide feedback on the program."		
**6a-i) Online questionnaires: describe if they were validated for online use and apply CHERRIES items to describe how the questionnaires were designed/deployed**		
**6a-ii) Describe whether and how "use" (including intensity of use/dosage) was defined/measured/monitored**		
**6a-iii) Describe whether, how, and when qualitative feedback from participants was obtained**		
**6b) CONSORT: Any changes to trial outcomes after the trial commenced, with reasons**		
"The study was conducted at the University Medical Center Hamburg-Eppendorf (Germany)."		
**7a) CONSORT: How sample size was determined**		
**7a-i) Describe whether and how expected attrition was taken into account when calculating the sample size**		
**7b) CONSORT: When applicable, explanation of any interim analyses and stopping guidelines**		

"Psychopathological self-rating questionnaires were assessed at the baseline and post assessments. The BDI-II [49] served as the primary outcome. Secondary outcomes included changes in self-esteem and quality of life as well as the participants' subjective evaluation of MOOD.<br>Beck Depression Inventory-II<br><br>The BDI-II [49] was used to assess depressive symptom severity over the previous 2 weeks. The self-rating questionnaire comprises 21 items; for each item, the participant is asked to evaluate the severity of the symptom on a rating scale from 0 to 3, with higher scores indicating more severe depressive symptoms. An overall score of 0 to 13 indicates minimal depression, 14 to 19 indicates mild depression, 20 to 28 indicates moderate depression, and 29 to 63 suggests severe depression. The internal consistency of the BDI-II ranges from 0.79 to 0.90 [50].<br>Patient Health Questionnaire–9—Depression Module<br><br>Change in depressive symptoms was also assessed with the Patient Health Questionnaire–9 (PHQ-9) [51]. The PHQ-9 is a self-rating questionnaire that comprises 9 items on depression, which can be answered on a 4-point rating scale ranging from 0= not at all to 3= nearly every day. Sum scores can range from 0 to 27 with the following classifications: none or minimal (0-4), mild (5-9), moderate (10-14), and severe (15-27) depressive symptoms. Results of the questionnaire can assist in determining a diagnosis of MD according to Diagnostic and Statistical Manual of Mental Disorders, 4th Edition, criteria. Its internal consistency ranges from 0.86 to 0.89 [50].<br>Rosenberg Self-Esteem Scale<br><br>The Rosenberg Self-Esteem (RSE) scale [52] was used to assess self-esteem. The scale comprises 10 statements regarding self-esteem. Participants are instructed to rate how much they agree with the statements on a 4-point Likert scale from strongly agree to strongly disagree. Its internal consistency ranges from 0.77 to 0.88. In its original form, higher scores reflect less self-esteem; however, in our study we used a reversed rating scale such that higher scores reflect more self-esteem.<br>World Health Organization Quality of Life–Abbreviated Version<br><br>The WHO Quality of Life–abbreviated version (WHOQOL-BREF) assesses quality of life [53]. It is a short version of the WHOQOL-100, with 26 items. The questionnaire contains 4 different types of 5-point rating scales that ask the participant how much, how complete, how often, how good, or how satisfied he or she felt over the previous 2 weeks. The questionnaire has 4 subscales: physical health, psychological, social relations, and environment. The WHOQOL-BREF has an internal consistency of 0.70 [54].<br>University of Rhode Island Change Assessment<br><br>The URICA scale is a measure of willingness to change [34] and was used in the baseline survey. It comprises 32 items representing 4 phases of change: precontemplation, contemplation, action, and maintenance. In this study, a total of 9 items were used, selected from the subscales of precontemplation, contemplation, and action. The internal consistency is 0.83, and the reliability of the test-retest lies between 0.63 and 0.75. Answers could be given on a 5-point Likert scale ranging from 1 (strong disagreement) to 5 (strong agreement). In addition, a single item on expectation regarding the treatment outcome was added: "At present, how successful do you think the MOOD self-help program will be?" Here, answers could be given on a 9-point Likert scale ranging from 1 (not successful at all) to 9 (very successful).<br>Subjective Appraisal<br><br>The subjective appraisal of the intervention was assessed with questions we generated, as well as with adaptions of the Fragebogen zur Patientenzufriedenheit ZUF-8 (questionnaire to measure patient satisfaction [55]). The internal consistency of the ZUF-8 ranges between 0.87 and 0.93. Items could be answered on a 4-point Likert scale ranging from totally disagree to totally agree. Open questions gave participants the opportunity to provide feedback on the program."		
**8a) CONSORT: Method used to generate the random allocation sequence**		
"Participants were randomly allocated into 1 of 2 conditions according to a randomization plan that was set up by the second author using the software Research Randomizer [36]."		
**8b) CONSORT: Type of randomisation; details of any restriction (such as blocking and block size)**		
"Block randomization was used to ensure balance between groups."		
**9) CONSORT: Mechanism used to implement the random allocation sequence (such as sequentially numbered containers), describing any steps taken to conceal the sequence until interventions were assigned**		
"As the study was conducted on the Web and the participants could actively enroll via Web-based registration, the allocation procedure differed from that in classical clinical trials, where allocation is performed by team members. On the basis of the date and time of completion of the baseline assessment, the participants were allocated to conditions following the randomization plan. The allocation rule was 1:1."		
**10) CONSORT: Who generated the random allocation sequence, who enrolled participants, and who assigned participants to interventions**		
"As the study was conducted on the Web and the participants could actively enroll via Web-based registration, the allocation procedure differed from that in classical clinical trials, where allocation is performed by team members. On the basis of the date and time of completion of the baseline assessment, the participants were allocated to conditions following the randomization plan. The allocation rule was 1:1. Participants who were allocated into the intervention group received an email containing information on the program and a link to the login Web page of MOOD as well as individual login data in the form of a code and a password. Participants in the CAU group received an email with the information that they would receive access to MOOD after completion of the post assessment."		
**11a) CONSORT: Blinding - If done, who was blinded after assignment to interventions (for example, participants, care providers, those assessing outcomes) and how**		
**11a-i) Specify who was blinded, and who wasn't**		
Blinding was not necessary as the study administrators did not have any information about the participants and "the participants could actively enroll via Web-based registration, the allocation procedure differed from that in classical clinical trials, where allocation is performed by team members. On the basis of the date and time of completion of the baseline assessment, the participants were allocated to conditions following the randomization plan."		
**11a-ii) Discuss e.g., whether participants knew which intervention was the "intervention of interest" and which one was the "comparator"**		
**11b) CONSORT: If relevant, description of the similarity of interventions**		
Not applicable as there was no active control group.		
**12a) CONSORT: Statistical methods used to compare groups for primary and secondary outcomes**		
"Intention-to-treat (ITT), per-protocol (PP), and frequent user analyses were conducted using IBM Statistics 25 software. The ITT sample comprised all participants who participated in the baseline assessment, whereas the PP sample comprised those who completed both the baseline and the post assessments and used the intervention at least once during the intervention period. Those who used MOOD at least once a week were considered frequent users. According to the Consolidated Standards of Reporting Trials guidelines for reporting RCTs, both ITT and PP analyses should be reported [56]. Although reporting ITT analyses might be considered as the standard analysis for clinical trials, the PP analyses provide an estimate of the true efficacy, as it only includes participants who completed the study and showed (partial) adherence. Missing values in the ITT analysis were imputed using an expectation-maximizing algorithm with 200 imputations. For each measure, we conducted ANCOVA for all samples (ITT, PP, and frequent user) with pre-post differences as the within-group factor, condition as the between-group factor, and baseline scores as the covariate to account for regression toward the mean [57]. In addition, an exploratory moderation analysis was conducted for the PP sample to identify possible moderators that affected differential symptom improvement for the 2 conditions (outcome measure: BDI-II difference scores) using the SPSS macro PROCESS by Hayes [58]."		
**12a-i) Imputation techniques to deal with attrition / missing values**		
"Missing values in the ITT analysis were imputed using an expectation-maximizing algorithm with 200 imputations."		
**12b) CONSORT: Methods for additional analyses, such as subgroup analyses and adjusted analyses**		
"In addition, an exploratory moderation analysis was conducted for the PP sample to identify possible moderators that affected differential symptom improvement for the 2 conditions (outcome measure: BDI-II difference scores) using the SPSS macro PROCESS by Hayes [58]."		
RESULTS		
**13a) CONSORT: For each group, the numbers of participants who were randomly assigned, received intended treatment, and were analysed for the primary outcome**		
"63 were randomized to the CAU condition and 62 to the MOOD condition (see the study flowchart in Figure 1)." In figure 1 and table 3, all relevant information can be found.		
**13b) CONSORT: For each group, losses and exclusions after randomisation, together with reasons**		
All relevant information can be found in the provided CONSORT flow diagram (figure 1).		
**13b-i) Attrition diagram**		
**14a) CONSORT: Dates defining the periods of recruitment and follow-up**		
"The first participant was included on May 8, 2018, and the last post assessment took place on July 22, 2018. "		
**14a-i) Indicate if critical "secular events" fell into the study period**		
**14b) CONSORT: Why the trial ended or was stopped (early)**		
Not applicable as the trial did not stop early or unintended.		
**15) CONSORT: A table showing baseline demographic and clinical characteristics for each group**		
All relevant information is provided in Table 2.		
**15-i) Report demographics associated with digital divide issues**		
All relevant information is provided in Table 2.		

**16a) CONSORT: For each group, number of participants (denominator) included in each analysis and whether the analysis was by original assigned groups**		
**16-i) Report multiple “denominators” and provide definitions**		
All relevant information can be found in Tables 2-6 and under the section "Results".		
**16-ii) Primary analysis should be intent-to-treat**		
**17a) CONSORT: For each primary and secondary outcome, results for each group, and the estimated effect size and its precision (such as 95% confidence interval)**		
All relevant information including effect sizes and confidence intervals are provided in table 3.		
**17a-i) Presentation of process outcomes such as metrics of use and intensity of use**		
**17b) CONSORT: For binary outcomes, presentation of both absolute and relative effect sizes is recommended**		
Not applicable as no binary outcomes are included.		
**18) CONSORT: Results of any other analyses performed, including subgroup analyses and adjusted analyses, distinguishing pre-specified from exploratory**		
Results of the moderation analysis are provided under the subsection "Moderation analysis" as well as in Figure 2 and Table 4.		
**18-i) Subgroup analysis of comparing only users**		
**19) CONSORT: All important harms or unintended effects in each group**		
Not applicable as no unintended effects occured.		
**19-i) Include privacy breaches, technical problems**		
**19-ii) Include qualitative feedback from participants or observations from staff/researchers**		
DISCUSSION		
**20) CONSORT: Trial limitations, addressing sources of potential bias, imprecision, multiplicity of analyses**		
**20-i) Typical limitations in ehealth trials**		
"Strengths and Limitations<br><br>To participate in the study, neither a (verified) diagnosis of depression nor a minimum severity of depression symptoms was necessary, which resulted in heterogeneity of depression levels. Therefore, on the one hand, a wider range of individuals with desire for treatment for depression was reached, regardless of whether they met the criteria of a diagnosis or not. On the other hand, it has been found that samples of severely depressed participants benefit more from low-intensity psychological interventions than samples of mildly depressed participants [32], which might be because those with severe depression have more room to improve. This finding, however, contrasts with results of Karyotaki et al [21] who found that self-guided internet-based interventions are effective regardless of symptom severity. Interestingly, within another study by Karyotaki et al [71], it was found that individuals with more severe baseline symptoms were more likely to improve than individuals with less severe baseline symptoms after treatment with guided internet-based interventions, which means that the findings are still ambiguous in this respect. Apart from this, broad inclusion criteria may result in a type I error and, thereby, lead to an underestimation of the intervention’s treatment potential. Another limitation might be the sample size in our study. Our power analysis was based on a medium effect, and we did not consider expected dropout in our sample size calculation, which could have led to an overestimation, as Karyotaki et al [21] only found a small effect (g=0.27) for self-guided interventions. To detect such a small effect, a larger sample would have been necessary. (...)"		
**21) CONSORT: Generalisability (external validity, applicability) of the trial findings**		
**21-i) Generalizability to other populations**		
**21-ii) Discuss if there were elements in the RCT that would be different in a routine application setting**		
**22) CONSORT: Interpretation consistent with results, balancing benefits and harms, and considering other relevant evidence**		
**22-i) Restate study questions and summarize the answers suggested by the data, starting with primary outcomes and process outcomes (use)**		
"In our study, we aimed to investigate whether the use of a new self-guided internet-based intervention called MOOD over a period of 6 weeks would lead to a significant reduction in depressive symptoms compared with a CAU group (that got access to MOOD after completion of the post assessment) in a sample of individuals who had previously and/or currently received therapy (most of the participants had also already received a different internet-based intervention).<br><br>In our analyses, we found no significant difference across groups in any outcome variable (for ITT, PP, and frequent users). Contrary to our hypothesis, the group that received MOOD over the intervention period did not significantly improve in depressive symptoms compared with the CAU group. Both groups significantly improved over time and, interestingly, the difference here was more pronounced for the CAU group for the primary outcome, which was the BDI-II. Both groups significantly improved over time in levels of self-esteem and quality of life, but this effect again was not different across groups. These findings are different from previous trials investigating the effectiveness of self-guided internet-based interventions."		
**22-ii) Highlight unanswered new questions, suggest future research**		
Other information		
**23) CONSORT: Registration number and name of trial registry**		
"Clinical Trial: ClinicalTrials.gov NCT03795480; http://clinicaltrials.gov/ct2/show/NCT03795480"		
**24) CONSORT: Where the full trial protocol can be accessed, if available**		
Not applicable as not available.		
**25) CONSORT: Sources of funding and other support (such as supply of drugs), role of funders**		
"The study was partly supported by a grant of the Swiss National Science Foundation to SW and SM (Grant Number 159384) and a fellowship of the Swiss National Science Foundation to SW (Number 177678). The study was not externally funded. It was financially supported by private donations to the working group and by overhead funds of the institution."		
**X26-i) Comment on ethics committee approval**		
**x26-ii) Outline informed consent procedures**		
**X26-iii) Safety and security procedures**		
**X27-i) State the relation of the study team towards the system being evaluated**		
